# The de novo* CACNA1A* pathogenic variant Y1384C associated with hemiplegic migraine, early onset cerebellar atrophy and developmental delay leads to a loss of Cav2.1 channel function

**DOI:** 10.1186/s13041-021-00745-2

**Published:** 2021-02-08

**Authors:** Maria A. Gandini, Ivana A. Souza, Laurent Ferron, A. Micheil Innes, Gerald W. Zamponi

**Affiliations:** 1 Department of Physiology and Pharmacology, Alberta Children’s Hospital Research Institute, Hotchkiss Brain Institute, Cumming School of Medicine, University of Calgary, Calgary, AB Canada; 2 Department of Medical Genetics and Alberta Children’s Hospital Research Institute, University of Calgary, Calgary, AB Canada

**Keywords:** Calcium channel, Migraine, Ataxia, Mutation, Gating, P/Q-type

## Abstract

*CACNA1A* pathogenic variants have been linked to several neurological disorders including familial hemiplegic migraine and cerebellar conditions. More recently, de novo variants have been associated with severe early onset developmental encephalopathies. *CACNA1A* is highly expressed in the central nervous system and encodes the pore-forming Ca_V_α_1_ subunit of P/Q-type (Cav2.1) calcium channels. We have previously identified a patient with a de novo missense mutation in *CACNA1A* (p.Y1384C), characterized by hemiplegic migraine, cerebellar atrophy and developmental delay. The mutation is located at the transmembrane S5 segment of the third domain. Functional analysis in two predominant splice variants of the neuronal Cav2.1 channel showed a significant loss of function in current density and changes in gating properties. Moreover, Y1384 variants exhibit differential splice variant-specific effects on recovery from inactivation. Finally, structural analysis revealed structural damage caused by the tyrosine substitution and changes in electrostatic potentials.

## Introduction

**Fig. 1:**
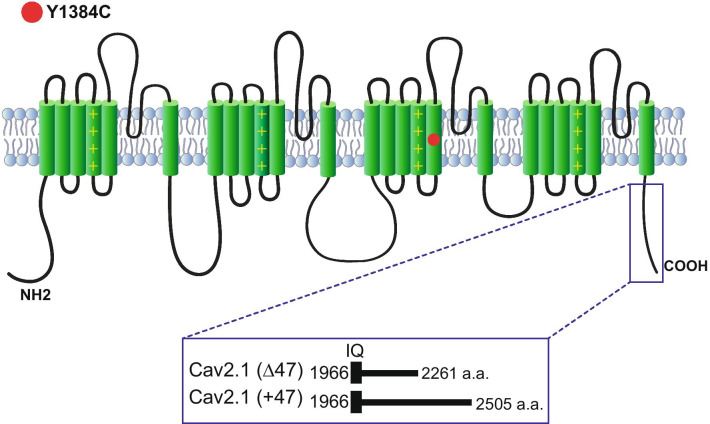
Ca_V_2.1 topology. Schematic representation showing the locus of the Y1384C substitution (red circle) on the secondary structure of the Ca_V_2.1α_1_ subunit. The Ca_V_2.1 terminus full-length C-terminal variant is produced by alternative splicing of exon 47

Voltage-gated Ca^2+^ (Ca_V_) 2.1 channels are the most abundant Ca_V_ channel in the mammalian brain where they are expressed in all brain structures with particularly high expression in the cerebellum [1, 2]. Ca_V_ channels are formed by the pore-forming Ca_V_α_1_ subunit and ancillary Ca_V_α2δ and Ca_V_β subunits. Ca_V_α_1_ encompasses 24 transmembrane α-helical segments divided into 4 domains (I–IV), each one with six segments (S1–S6). The voltage sensor is localized to the S4 segment of each domain, whereas S5 and S6 and their connecting loop form the ion conduction pathway of the channel (Fig. 1). Ca_V_2.1 channels couple efficiently to the vesicular release machinery and are involved in fast synaptic transmission [3, 4]. On postsynaptic neurons they play a role in Ca^2+^ signaling microdomains [5], neuronal excitability [6, 7] and gene expression (8).

Ca_V_2.1 channels undergo alternative mRNA splicing which can alter biophysical properties [9, 10]. The Ca_V_2.1 C-terminus region is highly divergent because of multiple alternative splicing sites, with inclusion of exon 47 producing a long version of the C-terminus (Fig. 1). C-terminal variations in human Ca_V_2.1 channels containing exon 47 represent 66% of the total expression in human cerebellum [11] and 79% in human cortex [12]. Due to their central role in neurotransmission, mutations in Cav2.1 channels are expected to impact synaptic transmission. Moreover, there are several reports where mutations in the Ca_V_2.1 gene (*CACNA1A*) cause several autosomal-dominant neurological disorders including familial hemiplegic migraine type 1, and cerebellar pathologies such as ataxia, progressive ataxia and early-onset cerebellar syndrome [13, 14]. Moreover, Ca_V_2.1 mutations have been associated with congenital ataxia, characterized by chronic cerebellar syndromes and acute symptoms of either episodic ataxia or hemiplegic migraine [13, 15]. Hemiplegic migraine (HM) is a rare type of migraine with aura associated with a transient motor weakness or hemiparesis [16]. HM can be familial (with an autosomal-dominant inheritance pattern) or sporadic (de novo mutations). Here, we describe the functional consequences of a de novo missense mutation c.4151A > G (p.Y1384C) in Cav2.1 channel activity. This mutation is located in the highly conserved transmembrane S5 of the third domain of the Ca_V_2.1α_1_ subunit (Fig. 1). The Y1384C variant was found in a patient with sporadic hemiplegic migraine, cerebellar atrophy and developmental disability, a phenotype previously reported in brief detail in this individual, as well as in another patient in the literature [17, 18]. Our functional studies reveal that this mutation causes a loss of function in the channel.

## Methods

### Patient and sequencing

The patient was ascertained and phenotyped through the clinical practice of one of the authors (AMI). The genetic sequencing was performed as previously reported [18].

### Molecular cloning

Wild-type (WT) human Cav2.1 α1 subunit (NM_000721.4) with or without exon 47 (+ 47 or Δ47), in pcDNA3.1, were kindly provided by Dr. Terrance Snutch (University of British Columbia). Site-directed mutagenesis was performed to insert the Y1384C mutation into both Cav2.1 (+ 47) and Cav2.1 (Δ47) channels by using the QuikChange site-directed mutagenesis kit (Agilent Technologies) instructions. Each construct was validated by sequencing of the whole coding region.

### Cell culture and recombinant Ca_V_2.1 channel expression

Human Embryonic Kidney tsA-201 cells were cultured in standard Dulbecco’s modified Eagle’s medium supplemented with 10% fetal bovine serum (heat inactivated) and 50 U/ml penicillin-50ug/ml streptomycin. Cells were kept at 37 °C in a humidified incubator with 5% CO_2_. Cells were transiently transfected using the calcium phosphate method with 3 µg of each plasmid encoding the pore-forming subunit Ca_V_2.1 (+ 47), Ca_V_2.1 (Δ47), Y1384C (+ 47) or Y1384C (Δ47), plus Ca_V_β_4_ and Ca_V_α_2_δ-1. For electrophysiology experiments, 0.5 µg of pEGFP was added to the transfection mix to identify and select transfected cells. A day after transfection cells were moved to an incubator at 30 °C and grown for 72 h post transfection before experiments.

### Electrophysiology

Electrophysiological recordings were performed at room temperature using whole cell configuration patch clamp with an Axopatch 200B amplifier (Molecular Devices). The external solution consisted of (in mM): 2 CaCl_2_, 137 CsCl, 1 MgCl_2_, 10 HEPES, 10 glucose (pH 7.4 adjusted with CsOH). The pipette solution contained (in mM): 130 CsCl, 2.5 MgCl_2_, 10 HEPES, 10 EGTA, 3 ATP-Mg, 0.5 GTP-Na (pH 7.4 adjusted with CsOH). Data acquisition was performed using pClamp11.03 software. Leak and capacitance components were subtracted on-line using a P/4 protocol. Currents were filtered at 5 kHz. Recordings were analyzed using Clampfit 11.03 and figures, fittings, and statistics (ANOVA) were made using GraphPad Prism 8.0. To ensure accurate comparisons, electrophysiological recordings alternated within the same day for all channel types.

### Recording protocols and data analysis

Ca_V_2.1 Ca^2+^ currents were recorded by applying 250 ms pulses ranging from − 60 to + 25 mV in 5 mV increments from a holding potential (hp) of − 100 mV. Current–density voltage relationships were obtained from the peak current divided by cell capacitance as described previously [19]. The I–V relationships were fitted with a modified Boltzmann equation: *I* = *Gmax* × *(Vm − Vr)/(1* + *exp(− (Vm − V*_*1/2*_*)/k))*, where *I* is the peak current, *Vm* is the membrane potential, *V*_*1/2*_ is the voltage for half activation, *Vr* is the reversal potential, and *k* is the slope factor. Activation curves were obtained by calculating conductance from I to V curves and plotting the normalized conductance (*G/Gmax*) as a function of membrane potential. Percentage (%) of inactivation was calculated as the percentage of current inactivated at 250 ms with respect to the peak current amplitude. Steady-state inactivation curves were obtained using depolarizations to − 5 mV for 140 ms following 5 s prepulses from − 100 to 0 mV at 10 mV increments from a holding potentiual of − 120 mV. Curves were constructed by plotting the normalized current (*I/Imax*) as a function of the prepulse potential and fitted with the equation *I/Imax* = *1/ (1* + *exp(− (Vm − V*_*1/2*_*)/k))*, where V_1/2_ is the voltage for half inactivation, and *k* is the slope factor. Recovery from inactivation was determined using a two pulse protocol. The first pulse (2 s) and second pulse (50 ms) were at 0 mV and separated by a varying interval ranging from 20 ms to 7.5 s. Traces were normalized to the maximum current during the first pulse for each sweep and plotted against time. Curves were fitted with a single exponential function. Window currents were plotted by using the values obtained from the fits of conductance and steady-state inactivation curves.

### Surface biotinylation

Surface biotinylation was performed as described previously [20]. Briefly, transfected cells were incubated on ice with HEPES-based saline solution (HBSS) for 15 min to stop protein trafficking. Surface proteins were then biotinylated for 1 h with 1 mg/ml of EZLink Sulfo-NHS-SS-Biotin (Thermo Scientific) in HBSS. The reaction was quenched with a solution of 100 mM Glycine in HBSS and cells were washed and lysed in a modified RIPA buffer (in millimolar: 50 Tris, 150 NaCl, 5 EDTA, 1% Triton X-100, 1% NP-40, 0.2% SDS, pH 7.4) for 45 min. Two mg of biotinylated proteins were purified using 100 µl of Neutravidin beads (Thermo Scientific) for 1.5 h at 4 °C. Biotinylated fractions and whole cell lysates were resolved by SDS-PAGE and analyzed by western blot using an anti-Ca_V_2.1α_1_ antibody (ACC-001, Alomone, 1:500) and anti-Na/K ATPase (1:5000, Abcam AB7671). Densitometric analyses were carried out using the ImageJ program (National Institutes of Health).

### Protein structural modeling

Homology models of full length Ca_V_2.1α_1_ subunits were generated using Phyre^2^ [21] using Cav1.1α_1_ as a template where 66% of the residues were modelled with > 90% of confidence. Missense mutation analysis was performed using Missense3D [22]. Models of the wild type and mutated of transmembrane segments S5 and S6 as well as the S5–S6 linker domain III were obtained using Phyre^2^ and electrostatic potentials were applied based on the full nonlinear solution of the Poisson–Boltzmann equation (23) using the Swiss-PdbViewer software.

### Statistical analysis

All error bars reflect standard errors. One-way analysis of variance (ANOVA) and Tukey’s multiple comparison test with a single pooled variance test was performed for multiple comparisons. Significance was set at 0.05. Significance was established as follows: *p < 0.05, **p < 0.01, *** p < 0.001, **** p < 0.0001.

## Results

### Clinical characteristics

The patient is a now 31 year old male. Some basic elements of his history were previously reported ([18], patient 18). In brief, he was born at 37 weeks gestational age to a 32 year old G1P0 mother. Apgar scores were 5 and 8. Hypotonia was present from the newborn period and he first presented to attention in Clinical genetics at nearly 3 years of age for evaluation of his developmental delay and hypotonia. Early computerized tomography (CT) of the brain was reported as normal. Subsequent magnetic resonance imaging (MRI) scanning did reveal evidence of generalized cerebellar atrophy. Initial genetic investigations including karyotype and Fragile X testing were normal.

At age 15 he presented back to genetics in the context of developing episodes consistent with hemiplegic migraine. As this time, given his constellation of features which include cerebellar ataxia, nystagmus, cranial nerve palsies, and a decline in IQ, a diagnosis of an atypical and severe early onset form of familial hemiplegic migraine was suspected. Genetic sequencing revealed the de novo missense change Y1384C in *CACNA1A*. In the 15 years since his genetic diagnosis, he has continued to follow a complex clinical course with cognitive decline, behavioural disturbances, disrupted sleep and yet no evidence of confirmed seizures. His care has been refractory to multiple medications.

One previous patient has also been reported in the literature with the same de novo variant. This individual was 33 years old at the time of publication [17] and has a similar history to the patient above with an uneventful birth history, cerebellar ataxia, nystagmus, global developmental delay, intellectual disability (estimated IQ of 40) and development of infrequent hemiplegic migraines beginning in childhood. These patients, and this specific variant, have been identified in reviews of FHM as being a variant of unique consequences, with an atypically severe phenotype [24].

### The Y1384C mutation decreases Ca^2+^ current density

**Fig. 2:**
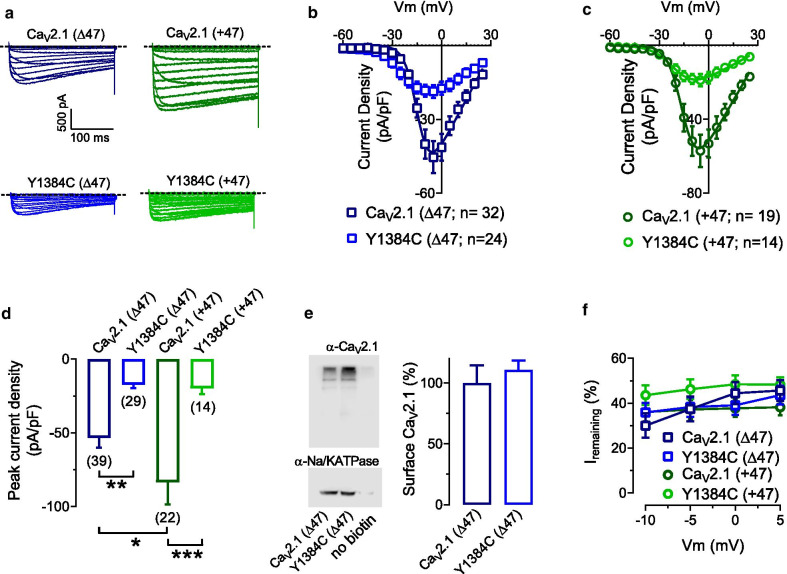
Average current densities of Ca_V_2.1 (Δ47), Y1384C (Δ47), Ca_V_2.1 (+ 47) and Y1384C (+ 47) channels. **a** Representative whole cell Ca^2+^ current traces recorded in response to depolarizing steps from − 60 mV to + 30 mV from a holding potential of − 100 from tsA-cells expressing either Ca_V_2.1(Δ47), Y1384C (Δ47), Ca_V_2.1(+ 47) or Y1384C (+ 47) α1 subunit with Ca_V_β_4_ and Ca_V_α_2_δ-1. **b** Average current density–voltage relationships for cells expressing Ca_V_2.1(Δ47) and Y1384C (Δ47) channels. **c** Average current density–voltage relationships for cells expressing Ca_V_2.1(+ 47) and Y1384C (+ 47) channels. **d** Average peak current density recorded from cells expressing Ca_V_2.1 (Δ47), Y1384C (Δ47), Ca_V_2.1 (+ 47) and Y1384C (+ 47) channels. **e** Left: Representative blot of Ca_V_2.1 (Δ47) and Y1384C (Δ47) channels surface expression (top blot) and Na/K ATPase surface expression (bottom blot). Right: Quantification of plasma membrane Ca_V_2.1 (Δ47) and Y1384C (Δ47) channel expression normalized by Na/K ATPase cell surface expression. Data are from 3 independent experiments. **f** Percentage of remaining current at the end of the 250 ms test pulse with respect to the peak current amplitude. The numbers in parentheses represent the numbers of cells recorded. Asterisks denote significance *0.05, **0.01, ***0.001 levels (One-way ANOVA, and Tukey’s multiple comparison test, with a single pooled variance test)

To assess the functional effects of the missense mutation p.Y1824C, the mutation was introduced into the human Ca_V_2.1 channel (splice isoforms + 47 and Δ47), and whole cell Ca^2+^ currents from transiently transfected cells (Ca_V_2.1 or Y1384C (+ 47 or Δ47) with Ca_V_β_4_ and Ca_V_α_2_δ-1) were recorded using the patch-clamp technique. Representative whole-cell Ca_V_2.1 current recordings are shown in Fig. 2a. Figure 2b, c show the average current density–voltage relationships (peak current amplitude normalized by *Cm*) in response to 250 ms depolarizations from a holding potential (*V*_*h*_) of − 100 mV. The current densities of the Y1384C mutant were consistently smaller across a range of voltages when compared to WT Ca_V_2.1channel controls (Fig. 2b, c). In agreement with previous findings Ca_V_2.1 (+ 47) channels exhibited larger peak current densities than Ca_V_2.1 (Δ47) ([10]; Fig. 2d). Y1384C mutants exhibited dramatically reduced peak current density indicating a marked loss of function (Fig. 2d). These data indicate that Y1384C mutants are either functionally inhibited or that they exhibit reduced trafficking to the cell surface. To discriminate between these two possibilities, we performed cell surface biotinylation experiments with Ca_V_2.1 (Δ47) and Y1384C (Δ47) channels. As shown in Fig. 2e, the mutation did not affect the membrane expression of the channel, indicating that the decrease in current density is due to a biophysical effect. To determine if the Y1384C mutation influences inactivation kinetics, we measured the percentage of current inactivated at the end of a 250 ms test pulse. The mutation did not affect this parameter (Fig. 2e).

### ***The Y1384C mutation modifies channel gating properties of Ca***_***V***_***2.1 splice isoforms***

**Fig. 3:**
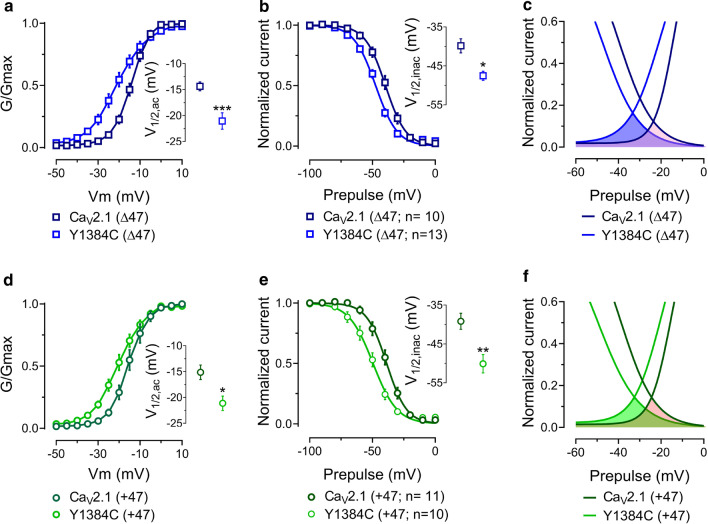
Gating properties of Ca_V_2.1 (Δ47), Y1384C (Δ47), Ca_V_2.1 (+ 47) and Y1384C (+ 47) channels. **a** Voltage-dependence of activation for cells expressing Ca_V_2.1 (Δ47) and Y1384C (Δ47) channels. Inset: mean half-activation potential values. **b** Voltage-dependence of steady-state inactivation for Ca_V_2.1 (Δ47) and Y1384C (Δ47) channels. Inset: mean half-inactivation potential values. **c** Window current of Ca_V_2.1 (Δ47) and Y1384C (Δ47) variants. Conductance and steady-state inactivation curves were obtained using the values from the fit with Boltzmann functions. **d** Voltage-dependence of activation for cells expressing Ca_V_2.1 (+ 47) and Y1384C (+ 47) channels. Inset: mean half-activation potential values. **e** Voltage-dependence of steady-state inactivation for Ca_V_2.1 (+ 47) and Y1384C (+ 47) channels. Inset: mean half-inactivation potential values. **f** Window current of Ca_V_2.1 (+ 47) and Y1384C (+ 47) variants. The numbers in parentheses represent the numbers of cells recorded. Asterisks denote significance *0.05, **0.01, ***0.001 levels (One-way ANOVA and Tukey’s multiple comparison test, with a single pooled variance test)

To further study if Y1384C mutation had an effect on the functional properties of Ca_V_2.1 variants, we analyzed the voltage-dependence of activation (Fig. 3). Slope factors were significantly increased in both splice variants in the presence of the mutation (Ca_V_2.1 (Δ47): − 3.68 ± 0.15 n = 39, Y1384C (Δ47): − 6.29 ± 0.51 n = 29, p < 0.0001; Ca_V_2.1 (+ 47): − 3.35 ± 0.23 n = 22, Y1384C (+ 47): − 6.18 ± 0.50 n = 14, p < 0.0001; ANOVA). Y1384C (Δ47) exhibited a shift of ~ 6.6 mV to more hyperpolarized potentials on its mean half-activation potential compared with Ca_V_2.1 (Δ47) (Fig. 3a), and a similar effect was seen with Y1384C (+ 47) (Fig. 3d and Table 1), indicating a gain of function, that is however, offset by the reduced current densities reported in Fig. 2. Interestingly, several *CACNA1A* mutations located in the Cav2.1 channel pore (S5, S6 and linker) also mediate a shift to more hyperpolarized voltages and enhanced channel open probabilities [25, 26].

**Table 1:** Biophysical properties of WT and mutant channels

	Ca_V_2.1 (Δ47)	Y1384C (Δ47)	Ca_V_2.1 (+ 47)	Y1384C (+ 47)
V_1/2, act_ (mV)	− 14.37 ± 0.85 n = 39	− 21.04 ± 1.57*** n = 29	− 15.14 ± 1.40 n = 22	− 21.13 ± 1.39* n = 14
*k*	− 3.68 ± 0.15	− 6.29 ± 0.51****	− 3.35 ± 0.23	− 6.18 ± 0.50****
V_1/2, inac_ (mV)	− 39.85 ± 1.80 n = 10	− 47.54 ± 1.16* n = 13	− 39.18 ± 2.07 n = 11	− 50.10 ± 2.36** n = 10
*k*	7.56 ± 1.75	8.50 ± 2.14	7.17 ± 1.40	8.75 ± 1.67

One-way analysis of variance (ANOVA) and Tukey’s multiple comparation test, with a single pooled variance test was performed for multiple comparisons. Significance was established as follows: *p < 0.05, **p < 0.01, ***p < 0.001, ****p < 0.0001

To study if Y1384C mutation influences channel availability, the voltage dependence of inactivation was evaluated using 5-s pre-pulses depolarizations from − 100 to 0 mV preceding a 140-ms test potential to − 5 mV. We observed a hyperpolarizing shift of the mean-half inactivation potential of 7.7 mV for Y1384C (Δ47; Fig. 3b and Table 1) and 10.9 mV for Y1384C (+ 47; Fig. 3e and Table 1) without any changes in the slope factor (Table 1). Collectively, these data indicate that the Y1384C mutation produces significant alterations of Ca_V_2.1 channels gating, by altering both activation and inactivation of the Ca_V_2.1 (Δ47) and Ca_V_2.1 (+ 47) channels. Since both activation and inactivation curves were shifted in Y1384C channels, we analyzed the window current of Ca_V_2.1 channels. We observed an increase in the window current generated by Y1384C channels (Fig. 3c, f). The Y1384C (Δ47) variant showed a 41% increase in the area under the curves whereas Y1384C (+ 47) variant exhibited a 21% increase. In addition, both mutants exhibited a hyperpolarized shift of the peak-voltage of the window current compared to their Ca_V_2.1 control channel (Y1384C (Δ47): 9 mV and Y1384C (+ 47): 7.5 mV). Altogether, these sets of data indicate that Y1384C mutants exhibit a higher persistent activity which can be translated to a greater Ca^2+^ current over a physiologically relevant membrane potentials near the resting potential.

### *Y1384C (Δ47) and Y1384C (*+ *47) mutants exhibit differential effects of recovery from inactivation*

**Fig. 4:**
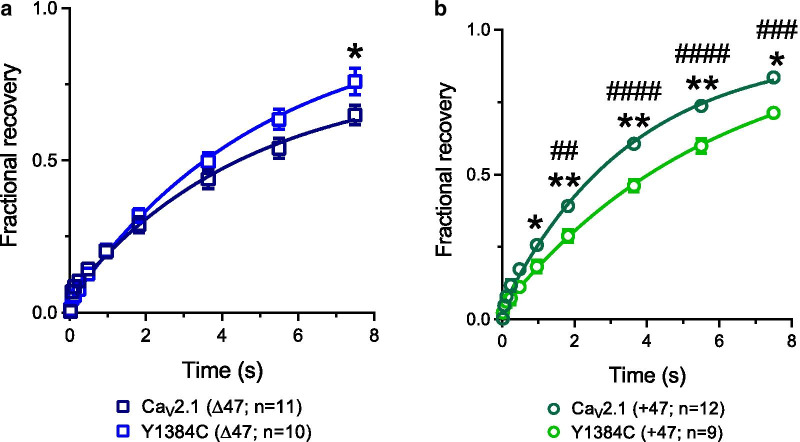
Recovery from inactivation of Ca_V_2.1 (Δ47), Y1384C (Δ47), Ca_V_2.1 (+ 47) and Y1384C (+ 47) channels. **a** Fractional recovery from inactivation of Ca_V_2.1 (Δ47), Y1384C (Δ47) channels. **b** Fractional recovery from inactivation of Ca_V_2.1 (+ 47), Y1384C (+ 47) channels. The numbers in parentheses represent the numbers of cells recorded. Symbols indicate significant difference relative to wild type of the same splicing isoform. Number signs indicate significant difference between Ca_V_2.1 isoforms. Asterisks and number signs denote significance *0.05, **0.01, ^##^0.01, ^###^0.001, ^####^0.0001 (One-way ANOVA and Tukey’s multiple comparison test, with a single pooled variance test)

To determine the time constant of fractional recovery from inactivation we used a two pulse protocol separated by a varying intervals ranging from 20 ms to 7.5 s and fitted with a single exponential function (Fig. 4). The Y1384C mutation did not modify the time constant of recovery from inactivation (Ca_V_2.1(Δ47): 5.84 ± 0.97 s, n = 11; Y1384C(Δ47): 6.33 ± 1.08 s, n = 10; Ca_V_2.1 (+ 47): 3.70 ± 0.23 s, n = 12 and Y1384C (+ 47): 6.19 ± 0.63 s, n = 9). As reported previously [12], Ca_V_2.1 (Δ47) and (+ 47) present a different fractional recovery (from 1.8 s to 7.5 s). Interestingly, the Y1384C (+ 47) variant exhibited a smaller fraction of channels recovered than Ca_V_2.1 (+ 47) channels at several time points (Fig. 4b). On the other hand, the Y1384C (Δ47) variant was not affected in this manner.

### Structural modeling of the Y1384 variant

**Fig. 5:**
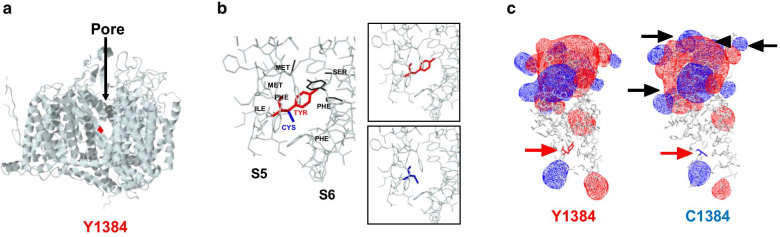
Ca_V_2.1 structural model. **a** Most probable 3D side model of the Ca_V_2.1α_1_ subunit. Position 1384 is shown in red. **b** Close up of the 1384 locus. Tyrosine1384 is shown in red and Cysteine 1384 in blue. In black are the changes caused by the substitution Y1384C. Insets show Tyrosine (top) or Cysteine (bottom) models. **c** Electrostatic potential distribution in transmembrane segments IIIS5 and IIIS6 and the connecting linker of Ca_V_2.1α_1_ (left) and Y1384C (right). The electrostatic potential of the analyzed region is shown in red (negative) and blue (positive). Red arrows denote the location of the mutation. Black arrows show changes in electrostatic potential

The Y1384C mutation is located in the transmembrane segment 5 of the third domain of the Ca_V_2.1α_1_ subunit. To understand how a change from tyrosine to cysteine can modify channel biophysical properties, we generated a homology model of Ca_V_2.1α_1_ (Fig. 5a). This model was used for structural analysis, revealing that the tyrosine substitution by cysteine leads to the expansion of cavity volume by 166.752 Å^3^, and this increase is consistent with structural damage. Figure 5b shows the modifications (black) of some amino acids positions when tyrosine (red) is mutated to cysteine (blue). Finally, we wanted to investigate if the channels have electrostatic potential changes because of the presence of the Y1384C mutation. For this purpose, we obtained the model of domain IIIS5 and IIIS6 and the interspersed extracellular loop in WT and mutant conditions and generated an electrostatic potential map. Figure 5c, shows apparent changes in the extracellular link (black arrows) which were probably allosterically induced by the structural damage caused by the amino acid substitution.

## Discussion

*CACNA1A* pathogenic variants have been classically associated with several disorders, including episodic ataxia type 2, spinocerebellar ataxia type 6 and hemiplegic migraine (familiar and sporadic [14, 27]). Only few cases of early onset ataxia, permanent ataxia, or early-onset cerebellar symptoms consistent with congenital ataxia have been associated with (de novo) *CACNA1A* pathogenic variants [13–15, 18, 28–32]. Our patient and the patient identified in Vahedi et al. [17] were two of the earliest reported examples of de novo developmental type disorders that are now increasingly recognized (see [33, 34]). Congenital ataxias (CA) represent 10% of nonprogressive infantile encephalopathies [35]. Patients with CA present neonatal hypotonia and motor delay, and during the first years of life progressive ataxia [35]. Here, we studied the *CACNA1A* p.Y1384C mutation found in two individuals with congenital ataxia, early onset cerebellar atrophy, sporadic hemiplegic migraine and intellectual disability [17, 18]. The symptoms in the patient reported here have been particularly challenging to manage. Given the emerging knowledge of the role in *CACNA1A* in various developmental encephalopathies, and the vision of provided precision care to rare disease patients, it is important to further understand the pathological consequences of individual rare variants.

Based on our modeling work, the Y1384C mutation is predicted to compromise channel function. Cav3 channels contain a cysteine in the position corresponding to tyrosine 1384 in Cav2 channels. This may perhaps explain why the Y1384C mutation leads to a hyperpolarizing shift in half activation voltage, leading to a gain of function. Based on our modeling, it is unlikely that this involves the formation of disulfide bridges. On the other hand, it is possible electrostatic potential changes induced by the mutation may affect permeation properties, leading to reduced currents. Remarkably, in a study where two different FMH-1 *CACNA1A* mutations were modeled (W1684R and V1696I which are both located in the S4 domain), the former caused electrostatic potential changes whereas the latter did not [36]. Patients bearing the W1684R mutation, but not the V1696I mutation, have been shown to exhibit cerebellar ataxia as part of their phenotype [37]. Y1384C channels present a loss of function in current density and a gain of function in gating properties, similar to other mutations located at the inner pore [14]. Of those two effects, the loss of function appears to dominate, as clearly seen from the current–density voltage relations in Fig. 2. Five other mutations associated with congenital ataxia located at the pore domain have been examined in functional studies (T666M, I1811L, D715E, ΔF1502 and V1396M [26, 38–41]). T666M (located at the selectively filter in domain III) is one of the most reported variants. This mutation induces a gain of function shift in the activation curve. Patients present normal to mild intellectual disabilities, mild or moderate ataxia and episodic coma. Normal posterior fossa structures to cerebellar atrophy restricted to the vermis or generalized have been reported [29, 42]. On the other hand, ΔF1502 (located at the S6 segment domain III) presents a reduction in current density, however when channels are stimulated with single or trains of action potentials, ΔF1502 exhibits an increase in Ca^2+^ influx after stimulation. Patients with this mutation have chronic cerebellar syndrome with acute HM events and epileptic seizures [40]. Although gain of function in gating parameters are consistent among these mutants, it is important to take into account that associated pathologies are a reflection of the balance of the expression of the mutations between excitatory and inhibitory circuits. Indeed, alternative splicing [11, 12], interaction with different ancillary subunits [43] and other regulatory and structural proteins [11, 44] can generate different pools of channels located in different neurons or even in the same synapse.

It is important to note that CACNA1A mutations can give rise to a wide spectrum of FHM1 severity. For example, the well documented R192Q variant is relatively mild whereas the S218L variant is severe and can lead to fatal edema [45], and in homozygous S218L mice, sudden unexpected death in epilepsy (SUDEP) is observed [46]. Interestingly, like with the Y1384C mutant, both variants have similar gain of function effects on half activation voltage [12]. They however differ in their kinetics of recovery from inactivation, with the S218L variant exhibiting more rapid recovery, similar to what we observed here. The amount of whole cell current remaining at the end of repetitive trains of action potentials was significantly depressed in the S218L variant compared to WT or R192Q channels [12], consistent with a loss of function. These types of loss of function mixed with gain of function characteristics complicate treatment strategies, and make it difficult to correlate severity of symptoms with observed biophysical changes. Such changes can potentially disrupt Ca^2+^ homeostasis in the cerebellum that can lead to congenital ataxia. For example, mutations on plasma membrane Ca^2+^ ATPases, abundantly expressed in Purkinje cells and granule cells, show a reduced capacity to extrude Ca^2+^, disrupting basal Ca^2+^ levels and/or Ca^2+^ signalling, resulting in altered synaptic efficiency and promoting hyperexcitability that gives rise to an ataxic phenotype [47–51]. Although there is no general mechanism established by which alterations of Ca^2+^ homeostasis in the cerebellum causes congenital ataxia, characterizing *CACNA1A*-linked mutations and their consequences in the cerebellar network may be important considerations for therapeutic interventions.

## Data Availability

All data generated or analyzed during this study are included in this published article.

## Abbreviations

FHM-1: Familial Hemiplegic Migraine type 1
HM: Hemiplegic migraine
CA: Congenital ataxia
CT: Computerized tomography
MRI: Magnetic resonance imaging
SUDEP: Sudden unexpected death in epilepsy
